# Development and validation of an interpretable machine learning model for predicting ADHD comorbidity in children with allergic rhinitis: a SHAP-based analysis

**DOI:** 10.3389/fpubh.2026.1837156

**Published:** 2026-09-23

**Authors:** Hexiu Yuan, Nengjing Wu

**Affiliations:** 1 Ganzhou Hospital-Nanfang Hospital, Southern Medical University (Ganzhou People’s Hospital), Ganzhou, China; 2 First Affiliated Hospital of Gannan Medical University, Ganzhou, China

**Keywords:** allergic rhinitis, attention-deficit/hyperactivity disorder, machine learning, neuro-immune crosstalk, SHAP

## Abstract

**Background:**

The co-occurrence of allergic rhinitis (AR) and attention-deficit/hyperactivity disorder (ADHD) presents significant clinical challenges in pediatrics, yet predictive tools capturing non-linear neuro-immune interactions are lacking.

**Methods:**

We developed an interpretable Extreme Gradient Boosting (XGBoost) model utilizing a hybrid feature selection pipeline on a cohort of 518 pediatric AR patients (255 with comorbid ADHD). Model performance was evaluated via an independent test set. Global and local interpretability was decoded using SHapley Additive exPlanations (SHAP).

**Results:**

A parsimonious subset of six predictors was identified. The XGBoost model achieved superior discriminative capacity (AUC = 0.878) compared to conventional linear baselines. SHAP topographical analysis revealed profound non-linear thresholds and uncovered a critical synergistic interaction: elevated ADHD risk was strongly correlated with patients presenting simultaneously severe allergic inflammation (Total IgE > 250 IU/mL) and nutritional deficiency (Vitamin D < 20 ng/mL). Furthermore, Decision Curve Analysis (DCA) demonstrated substantial net clinical benefit for triage application.

**Conclusion:**

We established a highly robust, interpretable machine learning algorithm capable of accurately stratifying ADHD risk among AR children. The identified neuro-immune synergistic phenotypes provide critical mechanistic insights and offer a practical decision-support tool for timely pediatric multidisciplinary interventions.

## 1 Introduction

Allergic rhinitis (AR) is one of the most prevalent chronic inflammatory disorders in pediatrics, characterized by persistent symptomatic burdens that significantly impair health-related quality of life (1). Over the past years, there has been a mounting body of epidemiological evidence that has shed light on a strong bidirectional relationship between atopic diseases and neurodevelopmental susceptibility, specifically attention-deficit/hyperactivity disorder (ADHD) (2, 3). The disproportionality of ADHD symptomatology has been consistently observed in severely affected AR patients of pediatric age as compared to healthy control populations (4). This common co-morbidity highlights an urgent issue in pediatric clinical practice where the concomitant cognitive dysfunction, i.e., inattention, impulsivity and sleep disturbance, often makes establishing a diagnosis challenging and therapeutic responses ineffective (5, 6).

Pathophysiological mechanisms which underlie this neuro-immune crosstalk are still not well understood, but new paradigms consider the contribution of systemic inflammation (7–9). It is postulated that neuroinflammation may be potentially linked to the perturbation of the blood–brain barrier associated with peripheral allergic cascades characterized by increased immunoglobulin E (IgE) and pro-inflammatory cytokines (10). At the same time, both immune dysregulation and dopaminergic dysfunction have been independently attributed to metabolic and lifestyle modulators, including serum Vitamin D deficiency and irregular sleep patterns (11, 12). Therefore, AR-ADHD comorbidity is inherently multifactorial, driven by complex, non-linear interactions between immunological, nutritional and environmental factors (13–15).

Nonetheless, these findings notwithstanding, the existing clinical risk assessment is greatly based on the traditional multi-variable logistic regression models (16–18). Although these conventional statistical models are useful in estimation of linear odds ratios, those models are limited by strict structural assumptions (19). They often fail to attain physiological thresholds, U shaped risk patterns and high-order synergistic interactions of predictors. An example of this is the compounded neurodevelopmental risk of concomitant allergic exacerbation and nutritional inadequacy which can be easily distorted in typical linear constructs (20). Therefore, a significant number of children at high risk do not get readily diagnosed, which precludes timely provision of multidisciplinary care.

In order to circumvent such analytical shortcomings, machine learning algorithms are introducing a potentially potent alternative, by self-inferring complex and multi-dimensional data structure without *a priori* parametric constraints (21). Nevertheless, the “black-box” nature of more sophisticated ensemble algorithms has historically made them less acceptable in transparent medical decision-making (22). The most recent addition of SHapley Additive exPlanations (SHAP) is a mathematical answer to this shortcoming, allowing granular quantification of the contribution of the individual features as well as visualization of non-linear clinical relationships (23).

Hence, the main aim of the research was to analytically compare the various machine learning frameworks and develop an optimal and highly explicable predictive model of ADHD risk within a pediatric AR cohort. Through the implementation of a stringent hybrid feature selection pipeline on standard pediatric evaluations, we aimed to distill a parsimonious subset of core clinical predictors. Then, we applied SHAP-based topological analysis on the most effective algorithm and we attempted to decipher the synergistic neuro-immune processes underlying this comorbidity and finally we applied this algorithmic knowledge to develop an effective stratified clinical triage instrument.

## 2 Materials and methods

### 2.1 Study population and ethical considerations

This retrospective cohort study included pediatric patients diagnosed with allergic rhinitis (AR) who were consecutively evaluated at the Department of Child Healthcare between January 2018 and January 2025. The diagnosis of AR was established according to the Allergic Rhinitis and its Impact on Asthma (ARIA) guidelines, while comorbid Attention-Deficit/Hyperactivity Disorder (ADHD) was confirmed by pediatric psychiatrists adhering strictly to the Diagnostic and Statistical Manual of Mental Disorders, Fifth Edition (DSM-5) criteria (24, 25). Patients with severe congenital anomalies, major psychiatric disorders other than ADHD, or severely incomplete core clinical data (missing rate > 10%) were systematically excluded. Furthermore, to eliminate confounding systemic inflammatory noise, patients diagnosed with severe or uncontrolled concurrent allergic diseases, such as active asthma or severe atopic dermatitis, were also strictly excluded from the cohort.

This study protocol was reviewed and approved by the Institutional Review Board of Ganzhou People’s Hospital (Approval No. 2026-065). All procedures were conducted in accordance with the ethical principles formulated in the Declaration of Helsinki. Written informed consent was obtained from the parents or legal guardians of all participating children prior to their enrollment in the study.

### 2.2 Clinical variables and feature engineering

A comprehensive panel of 15 baseline clinical indicators was extracted from electronic medical records, encompassing demographics, anthropometrics, birth history, laboratory indices, and lifestyle/environmental factors. The normality of continuous variables was assessed using the Shapiro–Wilk test. Continuous variables with normal distribution were summarized as the mean and standard deviation (SD) whereas non-normally distributed continuous variables (e.g., Total IgE, Screen Time) were summarized as the median and interquartile range (IQR). Absolute frequencies and percentages were used to show categorical variables. Univariate analysis was primarily conducted to evaluate baseline demographic comparability between the ADHD and non-ADHD groups, thereby identifying potential clinical confounding factors prior to the implementation of machine learning modeling.

The hybrid feature selection pipeline has been used in order to reduce the risk of model overfitting and identify the strongest predictors of the outcome across the high-dimensional clinical dataset. The feature selection was structurally designed into three parallel methodologies to reach a robust consensus. To strictly prevent data leakage and the inflation of downstream performance metrics, all feature selection procedures were executed exclusively on the 70% training set. First, a Least Absolute Shrinkage and Selection Operator (LASSO) regression, optimized via 10-fold cross-validation within the training data, was utilized to shrink non-contributory coefficients to zero. Second, the Boruta algorithm, a random forest wrapper, was implemented solely on the training cohort. Boruta evaluates feature importance by creating “shadow features”—randomly shuffled copies of the original variables—and rigorously compares the importance of original features against the maximum importance of these shadow counterparts, successfully discarding noise variables. Third, a Random Forest (RF) model was deployed to rank the variables based on the Mean Decrease Gini measure. Ultimately, an intersection of these three distinct methodologies yielded a consensus subset of core features, which was strictly utilized in definitive predictive modeling.

### 2.3 Machine learning pipeline and interpretability

The study cohort was randomly partitioned into a training set (70%) and an independent test set (30%) to ensure rigorous model validation. Strictly following the train-test split to prevent data leakage, data preprocessing was performed. For cases with sporadic missing values (<5%), the k-Nearest Neighbors (KNN) imputation algorithm was fitted solely on the training set and subsequently applied to both sets. Similarly, continuous clinical variables were standardized using the Z-score method, calculated as Z = (X − μ) / σ, where μ and σ were derived exclusively from the training population and applied to scale the test set, thereby eliminating dimensional biases without exposing future data. There were four implemented machine learning architectures, which included Logistic Regression (LR) as a linear control, and Random Forest (RF), Support Vector Machine (SVM), and Extreme Gradient Boosting (XGBoost) to detect non-linear interactions. Hyperparameter optimization was optimally performed with the use of grid search techniques on training data, where overfitting was inherently checked by the use of regularization methods (e.g., maximum tree depth, minimal node size, and stochastic subsampling).

Comprehensive assessment of model discriminative performance on the unseen test set was evaluated using the Area Under the Receiver Operating Characteristic Curve (AUC-ROC), Precision-Recall Curve (PRC), accuracy, sensitivity and specificity. To ensure a fair performance comparison, the optimal decision threshold for binary classification across all implemented machine learning models was uniformly determined by maximizing the Youden Index. Moreover, the model calibration was qualitatively evaluated using calibration plots and quantitatively verified using the Hosmer-Lemeshow goodness-of-fit test, while the clinical usefulness was determined using Decision Curve Analysis (DCA).

In order to address the issue of the black box nature of multidimensional ensemble algorithms, SHapley Additive exPlanations (SHAP) framework was used on the most successful model. SHAP summary beeswarm plots and clustering heatmaps were used to visualize global interpretability. SHAP partial dependence plots and an interaction strength matrix were created to decode synergistic non-linear mechanisms. Finally, Local Interpretable Model-agnostic Explanations-inspired (LIME) waterfall charts were applied to break down the personalized predictive paths of sample clinical cases. All statistical and calculation works were done in R software (version 4.4. 1, R Foundation of Statistical Computing, Vienna, Austria). A *p*-value of below 0.05 at both sides was taken to be statistically significant.

## 3 Results

### 3.1 Baseline characteristics and multidimensional data exploration of the study cohort

A total of 518 children with allergic rhinitis were enrolled in this study, comprising 255 cases with comorbid ADHD and 263 non-ADHD controls. The baseline demographic factors such as age, gender and birth history had no significant inter-group differences (Table 1). In addition, the levels of maternal education were also matched between the two sets of data (*p* = 0.460, Figure 1E), which provides an excellent basis to proceed with the predictive models.

**Table 1:** Baseline characteristics and multidimensional clinical phenotypes.

Variables	Overall (*N* = 518)	Control (No ADHD) (*n* = 263)	Case (ADHD) (*n* = 255)	*p*-value
Demographics and anthropometrics				
Age (years), mean (SD)	8.11 (1.92)	8.12 (1.96)	8.10 (1.88)	0.919
Gender, *n* (%)				0.649
Female	191 (36.9)	94 (35.7)	97 (38.0)	
Male	327 (63.1)	169 (64.3)	158 (62.0)	
BMI (kg/m ^2^), mean (SD)	17.28 (3.52)	17.54 (2.81)	17.02 (4.13)	0.095
Birth and feeding history				
Gestational age (weeks), median [IQR]	38.00 [37.00, 39.00]	38.00 [37.00, 39.50]	38.00 [37.00, 39.00]	0.620
Birth weight (kg), mean (SD)	3.19 (0.49)	3.20 (0.49)	3.18 (0.49)	0.668
Breastfeeding duration (months), median [IQR]	5.00 [3.00, 8.00]	5.00 [3.00, 8.00]	5.00 [3.00, 8.00]	0.975
Laboratory indicators				
Total IgE (IU/mL), Median [IQR]	190.50 [106.00, 302.50]	149.00 [84.00, 230.00]	251.00 [140.00, 360.50]	**<0.001**
Eosinophils (×10^9^/L), mean (SD)	0.36 (0.15)	0.36 (0.15)	0.36 (0.14)	0.945
Serum Vitamin D (ng/mL), mean (SD)	25.26 (6.89)	26.32 (6.25)	24.17 (7.36)	**<0.001**
Serum Ferritin (ng/mL), mean (SD)	39.95 (13.72)	41.18 (12.95)	38.68 (14.37)	**0.038**
Blood lead level (μg/dL), mean (SD)	3.48 (1.16)	3.37 (1.13)	3.60 (1.18)	**0.024**
Lifestyle and environmental factors				
Sleep duration (h/day), mean (SD)	8.96 (1.24)	9.09 (1.19)	8.82 (1.27)	**0.013**
Screen time (h/day), median [IQR]	1.65 [1.00, 2.70]	1.50 [0.80, 2.30]	1.80 [1.10, 3.00]	**<0.001**
Maternal education, *n* (%)				0.459
High School	153 (29.5)	73 (27.8)	80 (31.4)	
Undergrad	254 (49.0)	136 (51.7)	118 (46.3)	
Postgrad	111 (21.4)	54 (20.5)	57 (22.4)	
Family history of atopy, *n* (%)				**0.006**
No	129 (24.9)	79 (30.0)	50 (19.6)	
Yes	389 (75.1)	184 (70.0)	205 (80.4)	
History of otitis media, *n* (%)				0.521
No	333 (64.3)	173 (65.8)	160 (62.7)	
Yes	185 (35.7)	90 (34.2)	95 (37.3)	

SD, Standard Deviation; IQR, Interquartile Range; BMI, Body Mass Index; IgE, Immunoglobulin E; ADHD, Attention-Deficit/Hyperactivity Disorder.

Continuous variables with a normal distribution are presented as Mean (SD) and were compared using the Student’s *t*-test. Non-normally distributed continuous variables are presented as Median [IQR] and were compared using the Wilcoxon rank-sum test. Categorical variables are presented as counts (percentages) and were analyzed using Pearson’s Chi-square test or Fisher’s exact test, as appropriate. Bold *p*-values indicate statistical significance (*p* < 0.05).

**Figure 1:**
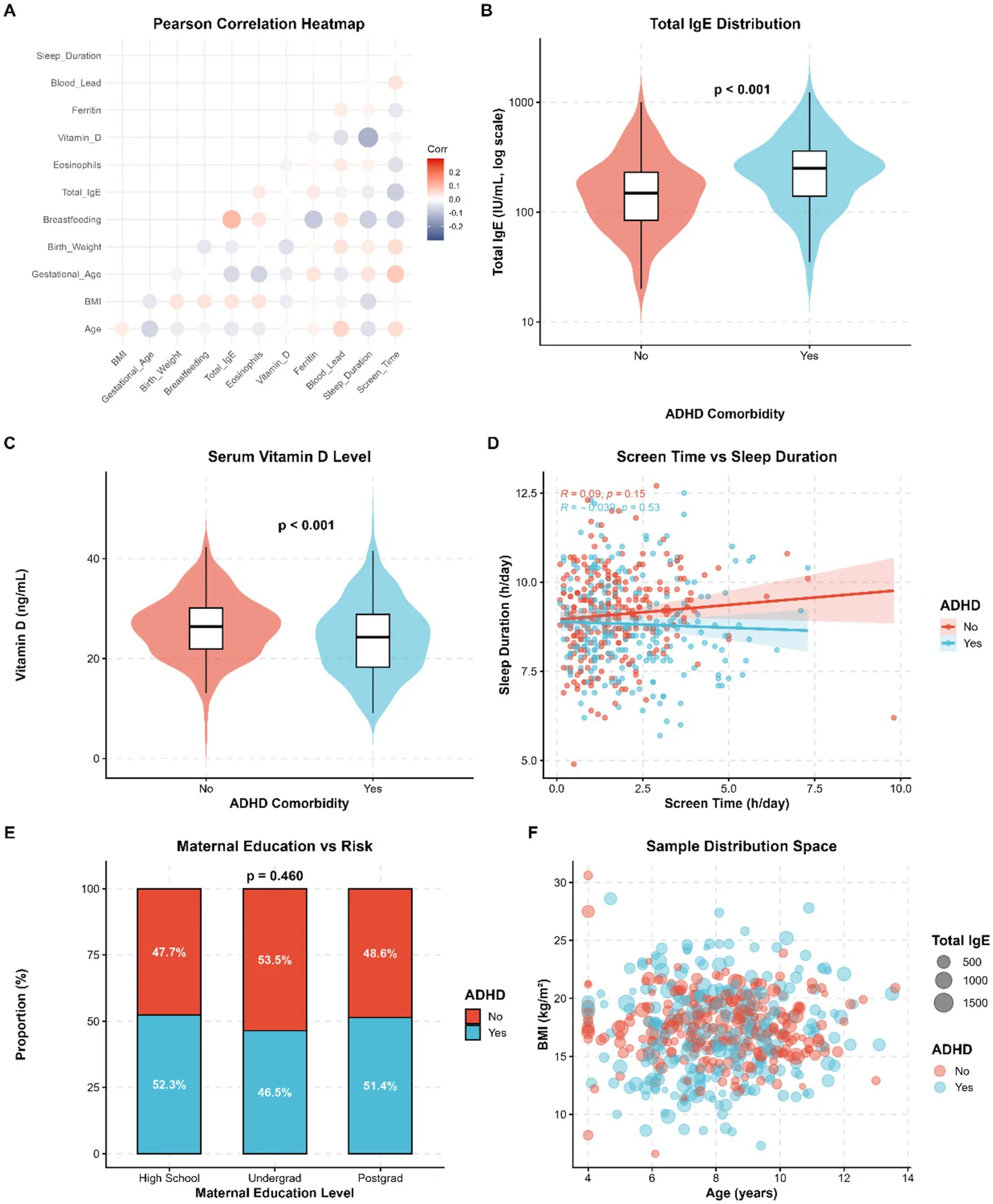
Baseline characteristics and multidimensional data exploration of the study cohort. **(A)** Pearson correlation heatmap of continuous clinical variables, indicating no severe multicollinearity. **(B,C)** Violin plots comparing Total IgE (log-scaled) and serum Vitamin D levels between the non-ADHD (Control) and ADHD (Case) groups. Differences were evaluated using the Wilcoxon rank-sum test and Student’s t-test, respectively. **(D)** Scatter plot with linear regression lines illustrating the relationship between screen time and sleep duration, stratified by ADHD comorbidity. **(E)** Stacked bar chart showing the proportion of ADHD risk across varying maternal education levels (analyzed by Chi-square test). **(F)** A multidimensional bubble plot depicting the sample distribution space, where the *x*-axis represents age, the *y*-axis represents BMI, bubble size indicates Total IgE levels, and colors represent actual clinical diagnosis.

Univariate analysis demonstrated that among the population with comorbidities, neuro-immunological and lifestyle parameters have changed significantly. ADHD group showed a significant increase of total IgE levels over the controls (251.00 vs. 149.00 IU/mL, *p* < 0.001; Table 1; Figure 1B). On the other hand, the serum Vitamin D levels were considerably depleted in the ADHD group (mean 24.17 vs. 26.32 ng/mL, *p* < 0.001; Figure 1C). There were other differences on environmental and behavioral variables where the ADHD group had a lower serum ferritin, increased blood lead levels, less sleep duration, increased screen time and a higher prevalence of familial atopy (all *p* < 0.05, Table 1).

A Pearson correlation matrix of the continuous clinical variables was used to evaluate the underlying data structure before the implementation of the machine learning method (Figure 1A). The lack of high values of the correlation coefficients (absolute r less than 0.20) excluded the existence of extreme multicollinearity. As an example, there was no significant linear dependence of the screen time and sleep duration in the case or control groups (Figure 1D), which indicated independent effect on the clinical outcome. Lastly, the intricate clinical phenotypes were represented in a multidimensional projection of the sample space (Figure 1F). Such spatial mapping intuitively showed that the increased levels of IgE, as represented by bubble size, were highly concentrated in the ADHD sub-population in different age and BMI groups, and this suggested a possible non-linear interaction between the immune system and neurodevelopment.

### 3.2 Robust feature selection for predictive modeling

Building upon the distinct clinical phenotypes identified in the univariable analysis, we further implemented a hybrid machine learning-based feature selection pipeline to ascertain the most robust predictors for ADHD comorbidity while mitigating the risk of model overfitting (Figure 2).

**Figure 2:**
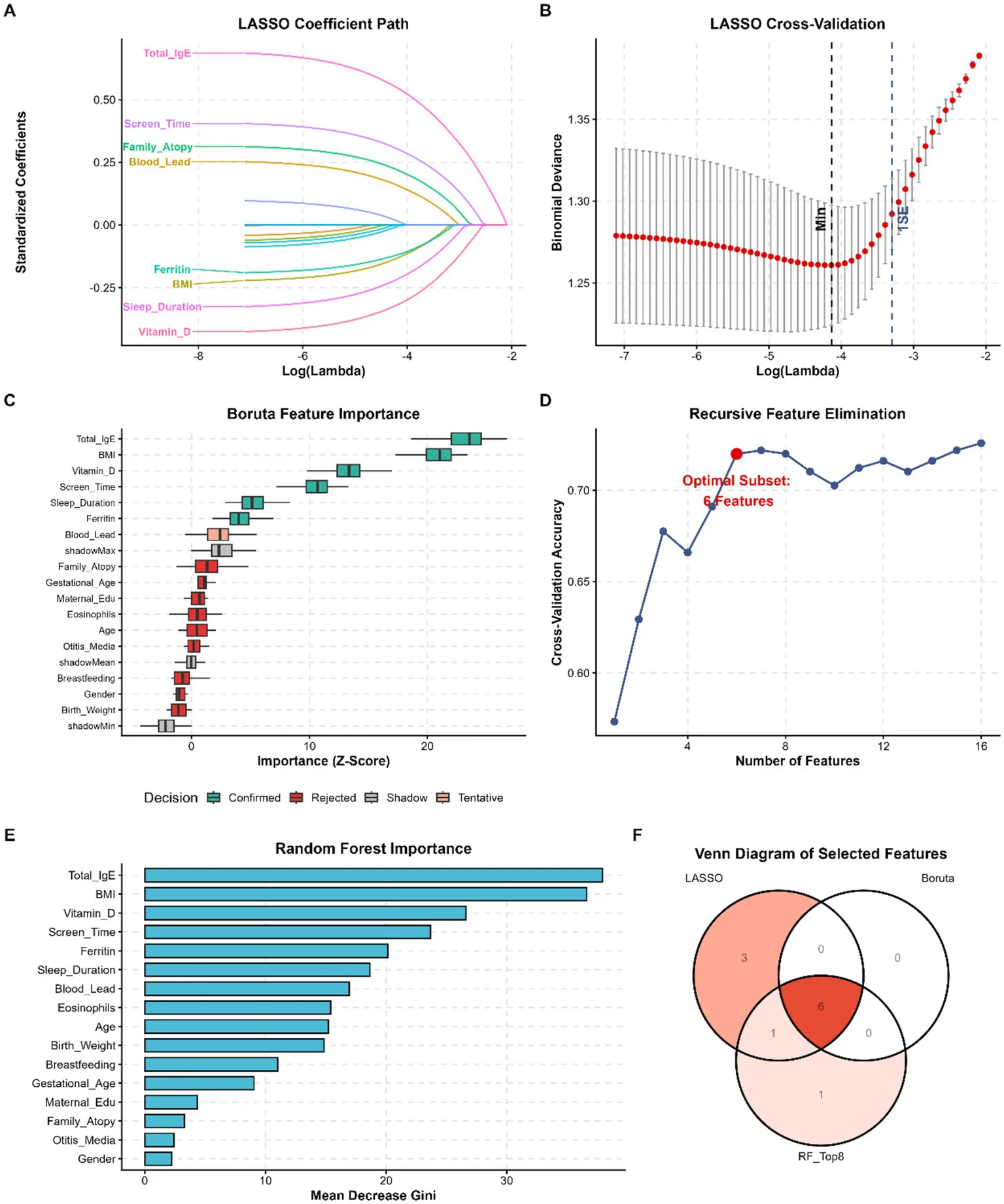
Hybrid feature selection process for predicting ADHD comorbidity. **(A)** The Least Absolute Shrinkage and Selection Operator (LASSO) coefficient path. **(B)** Binomial deviance evaluated via 10-fold cross-validation in the LASSO model. The black dashed line indicates the optimal lambda value yielding the minimum error. **(C)** Boruta feature importance algorithm, confirming robust predictors (green boxes) against randomized shadow features. **(D)** Recursive Feature Elimination (RFE) indicating that the cross-validation accuracy peaks at an optimal subset of 6 features. **(E)** Feature importance ranking derived from the Random Forest (RF) model based on Mean Decrease Gini. **(F)** A Venn diagram illustrating the intersection of the feature selection methods (LASSO, Boruta, and the top 8 RF features), resulting in 6 robust core predictors selected for subsequent model construction.

First, LASSO regression cross-validated with 10 folds on the standardized clinical variables was conducted. The coefficient path trajectory (Figure 2A) and binomial deviance curve (Figure 2B) proved that the minimum error of the model occurred at an optimal penalty parameter (lambda. min). In this strict penalization, coefficients of non-contributory variables were essentially shrunken to zero, leaving a small subset of salient features. At the same time, Boruta algorithm assessed feature importance repeatedly compared to randomized shadow attributes. This tree-based ensemble method decisively confirmed the significant predictive value of top-ranking indicators, particularly Total IgE, BMI, and Vitamin D (Figure 2C), which are considerably consistent with the univariable discrepancies of the baseline data.

Recursive Feature Elimination (RFE) was subsequently done in order to determine the best feature dimensionality. The cross-validation accuracy curve has shown that there was a steep upward slope at the very beginning, and at the point where the subset of only six features was selected, the addition of more variables did not create a significant performance improvement (Figure 2D). Moreover, the input of individual variables was ranked using the mean decrease Gini through a Random Forest (RF) model (Figure 2E), again highlighting the important effects of immunological and nutritional indices.

Finally, to combine the merits of the linear penalization and non-linear ensemble techniques, Venn diagram (Figure 2F) was drawn up. The intersection of feature subsets selected by LASSO, Boruta, and the top 8 RF features yielded a consensus of six characteristics: Total IgE, Vitamin D, BMI, Ferritin, Sleep Duration and Screen Time. This very stable core set was later used in the development and the comparison of the end predictive models.

### 3.3 Construction and comparative evaluation of predictive models

Leveraging the six core features identified through the hybrid selection pipeline, we developed and evaluated four distinct predictive algorithms: Logistic Regression (LR), Random Forest (RF), Support Vector Machine (SVM), and Extreme Gradient Boosting (XGBoost). Figure 3 shows that the models were trained on the main cohort and then tested on independent test set (to determine their generalizability and clinical discriminative capacity).

**Figure 3:**
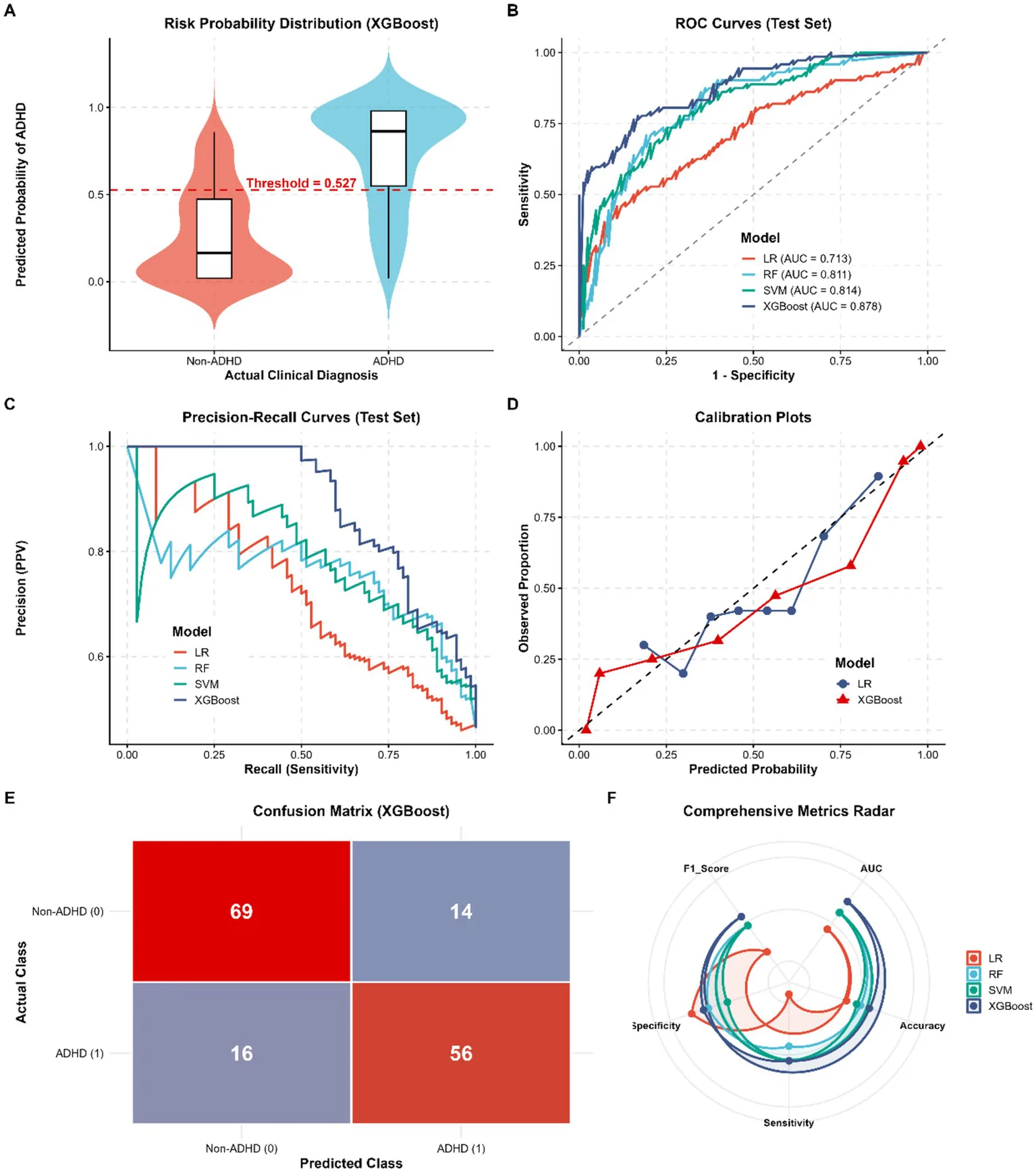
Predictive performance and comparative evaluation of machine learning models on the independent test set. **(A)** The predicted risk probability distribution of the optimal XGBoost model. The red dashed line denotes the optimal decision threshold (0.527) determined by the Youden Index, effectively stratifying actual ADHD cases from controls. **(B)** Receiver Operating Characteristic (ROC) curves comparing XGBoost with Random Forest (RF), Support Vector Machine (SVM), and Logistic Regression (LR). **(C)** Precision-Recall curves addressing the predictive reliability in the clinical cohort. **(D)** Calibration plots comparing the observed proportion of ADHD cases versus the predicted probabilities for XGBoost and LR. **(E)** The confusion matrix of the XGBoost model utilizing the optimal threshold, demonstrating a balanced sensitivity and specificity. **(F)** A radar chart comprehensively summarizing five key evaluation metrics (AUC, Accuracy, Sensitivity, Specificity, and F1-Score) across all constructed models.

However, the XGBoost model demonstrated better results in stratifying the test sample as shown in risk probability distribution plot (Figure 3A). Using the optimal decision threshold based on Youden Index of 0.527, the model was successful in distinguishing between ADHD and non-ADHD cases and the probability mass of cases actually obtained is largely concentrated above the decision threshold.

The comparison of the receiver operating characteristic (ROC) curves showed that XGBoost had the highest Area Under the Curve (AUC) of 0.878, which was better than the linear baseline LR model (AUC = 0.713) and the non-linear ones such as SVM (AUC = 0.814) and RF (AUC = 0.811) (Figure 3B). Detailed performance metrics, including the specific Youden Index-derived thresholds for each individual model, are comprehensively summarized in Supplementary Table S1. Since the clinical imperative was to reduce false discoveries, Precision-Recall (PR) curves were obtained that also supported the resilience of XGBoost with a high value of the positive predictive at a range of sensitivity rates (Figure 3C).

The calibration curve also showed that the expected probabilities using XGBoost were well-related to the observed prevalence of ADHD in the test group, which is likely to be feasible risk estimators (Figure 3D). This accuracy was statistically corroborated by the Hosmer-Lemeshow goodness-of-fit test, yielding a non-significant *p*-value (*p* = 0.315), thereby confirming that the model’s predicted probabilities did not significantly deviate from the actual clinical observations. With the use of the best above-mentioned threshold, the confusion matrix of the XGBoost model made a description of 69 true negatives, 56 true positives, 14 false positives, and 16 false negatives (Figure 3E). This had led to a balanced clinical performance having a sensitivity of 77.8 and specificity of 83.1.

An overview radar chart that compared the important evaluation metrics highlighted the benefits of the XGBoost architecture (Figure 3F). In particular, XGBoost showed a total accuracy of 80.6 percent and F1-score equal to 0.789, which means that it is effective in managing the interactions between the neuro-immune and lifestyle predictors that are complex and may not be linear, as determined during the selection phase 1.

### 3.4 Global interpretability and non-linear mechanism decoding via SHAP analysis

To decipher the internal decision-making process underlying the robust performance of the XGBoost model, we employed the SHapley Additive exPlanations (SHAP) algorithm for global and local interpretability (Figure 4). Mean absolute SHAP values were used to measure the contributions of all features in predictive output (Figure 4B). The most important predictor (mean |SHAP|) was found to be Total IgE (0.981), and sequentially BMI (0.854) and Vitamin D (0.601), confirming the initial predictors of the neuro-immune and metabolic factors found when the features were first screened.

**Figure 4:**
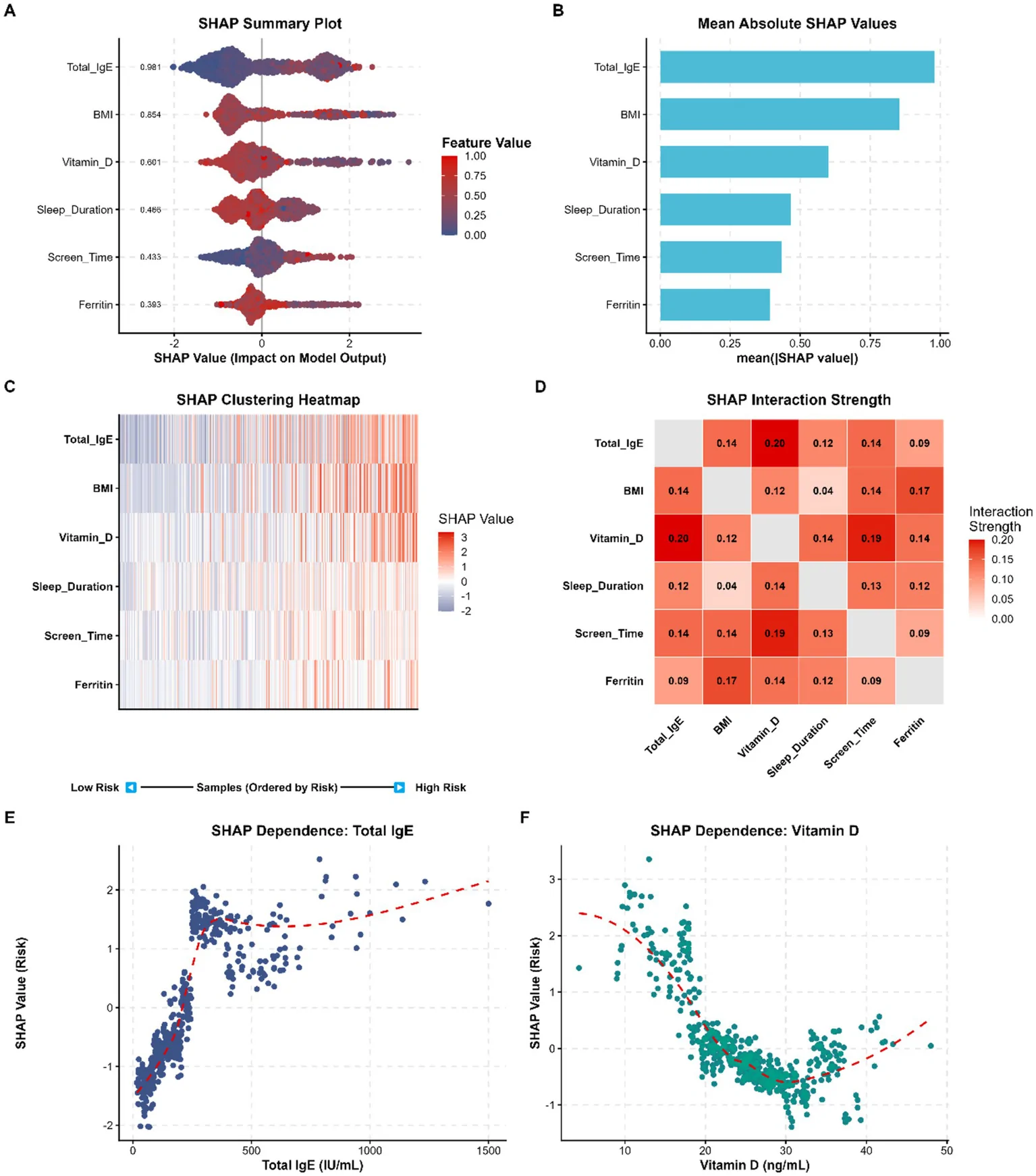
Global interpretability and non-linear mechanism decoding of the XGBoost model using SHAP analysis. **(A)** SHAP summary beeswarm plot ranking the top features. Each dot represents a single patient, with the color indicating the original feature value and the x-axis showing its impact on the model’s output (positive values denote increased risk). **(B)** Bar plot of mean absolute SHAP values, quantifying overall feature importance. **(C)** SHAP clustering heatmap aligning individual feature contributions for samples ordered by increasing predicted risk. **(D)** SHAP interaction strength matrix revealing potential synergistic effects among clinical indicators, with the strongest interaction observed between Total IgE and Vitamin D. **(E,F)** SHAP partial dependence plots for Total IgE and Vitamin D, with dashed lines generated by LOESS smoothing. The plots uncover critical non-linear physiological thresholds driving ADHD risk.

SHAP summary beeswarm plot allowed us to obtain a granular view of the impact that the magnitude of each feature has in individual risk prediction (Figure 4A). There was a clear directional trend in the highest-rated variables. In particular, high indicators of Total IgE (red dots) always corresponded to the positive SHAP values and were strongly correlated with higher ADHD comorbidity model predictions. On the other hand, the negative SHAP values predominantly produced by higher serum Vitamin D concentrations were associated with a protective profile on clinical outcomes.

In order to investigate the patterns of synergy of these predictors within the patient cohort, a heatmap of clusters of SHAP predictors ranked in ascending order based on individual predicted risk was created (Figure 4C). The heatmap clarified a prominent transition effect: the high-risk cohort (on the right) was characterized by a combination of positive SHAP contributions (red clusters) by the Total IgE and BMI as well as negative SHAP manifestations (blue clusters) by vitamin D deficiency. This finding led to a quantitative evaluation of feature interaction by a SHAP interaction strength matrix (Figure 4D). It was found that the strongest interaction happened between Total IgE and Vitamin D (interaction strength = 0.20), which indicates that a combination of strong allergic inflammation and nutritional insufficiency can cooperate to increase neurodevelopmental susceptibility.

Besides, SHAP partial dependence plots were created to decode the physiological non-linearities within the traditional regression models that cannot be modeled. The dependence plot of the Total IgE (Figure 4E) revealed a different type of threshold effect such that the risk of ADHD in relation to IgE level increased sharply as the level surpassed about 250 IU/mL, then leveled off. Equally, the protective effects of Vitamin D (Figure 4F) showed a non-linear pattern with the risk reduction effect obtained becoming minimal with a drop in concentrations to less than 20 ng/mL. These non-linear processes have a mechanistic cause behind a higher predictive power of the XGBoost model compared to the linear baseline already observed.

### 3.5 SHAP dependence and synergistic risk analysis

To further elucidate the complex interplay between the primary predictive features identified in the global interpretability phase, we conducted an in-depth SHAP dependence analysis (Figure 5). This method came with the possibility of visualizing the marginal effects and possible synergist effects between important variables.

**Figure 5:**
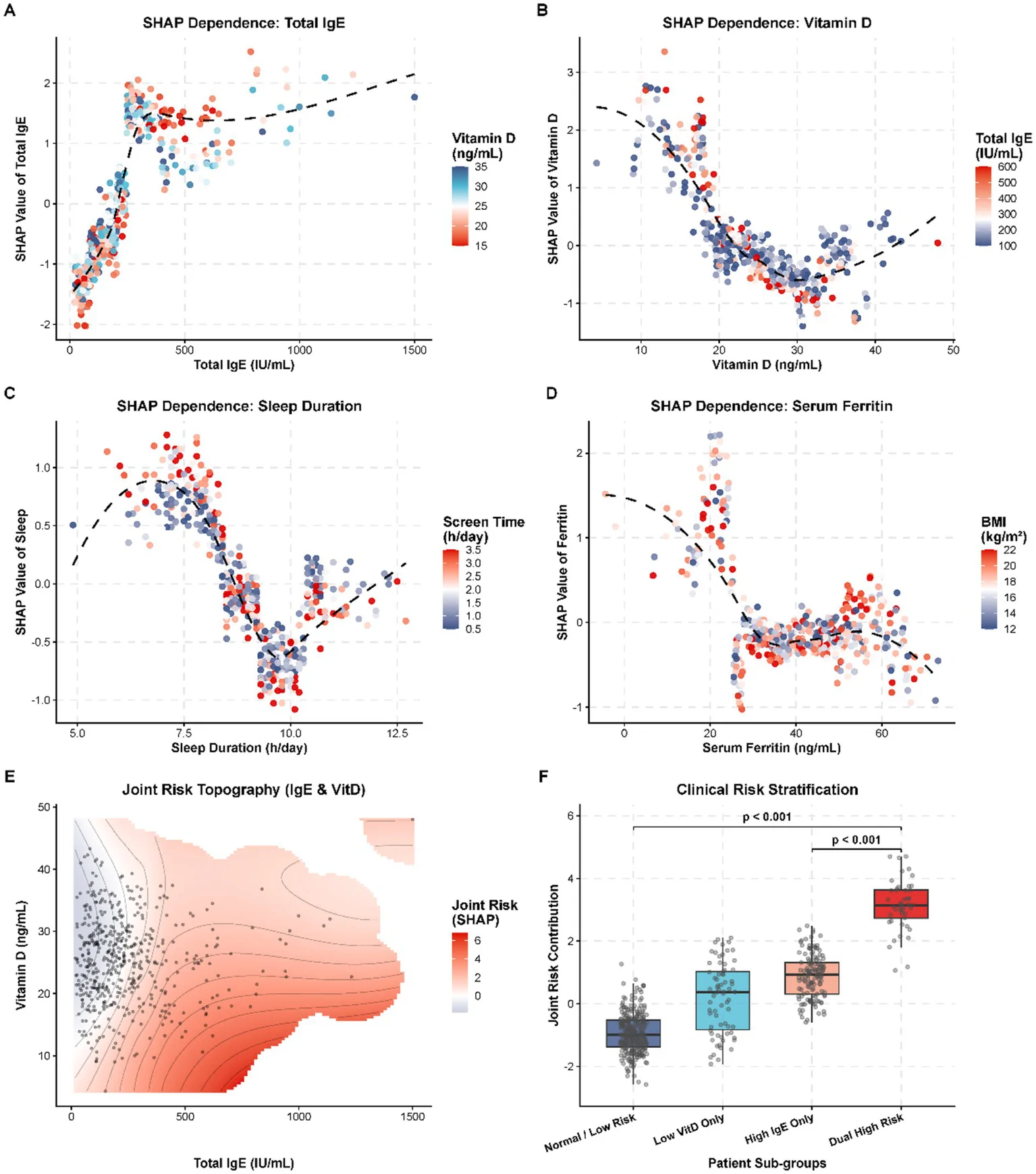
In-depth SHAP dependence and joint risk topography analysis. **(A–D)** SHAP dependence plots colored by a second interacting feature, vividly demonstrating interactive effects. **(E)** A joint risk topography contour map generated via LOESS surface interpolation. The deep red ‘storm eye’ in the bottom-right quadrant pinpoints the highest risk intensity synergistically driven by severe allergic inflammation (high IgE) and nutritional deficiency (low Vitamin D). **(F)** Clinical risk stratification dividing the cohort into four distinct sub-groups based on identified SHAP thresholds. The ‘Dual High Risk’ group exhibits a significantly exponentially compounded risk compared to singular risk groups (*p* < 0.001, Student’s *t*-test).

The SHAP dependence plot of Total IgE (Figure 5A) color-coded with the levels of Vitamin D showed a strong non-linear curve. The contribution or SHAP value to the risk of ADHD progressively increased with the increase in Total IgE with inflexion point at approximately 250–300 IU/mL. Remarkably, most of these risk-inducing levels in the high range of IgE were majorly found in people who were simultaneously under Vitamin D (denoted by red dots). The Vitamin D dependence pattern showed a reciprocity in the Vitamin D dependence plot, the plot (Figure 5B) displaying that the vitamin D concentrations below 20 ng/mL correlated with using steep gradient of SHAP values, which were strategically compounded in patients with high Total IgE (red dots). The findings indicate a high-degree of interaction, which implies a possibility that a severe allergic inflammation and nutritional inadequacy can exclusively demonstrate a synergistic association with an increased likelihood of neurodevelopment.

Other lifestyle and metabolic predictors showed similar non-linear correlations. The sleep duration had a u-shaped risk pattern, as both short sleep and excessive sleep increased the model output, and this effect was mediated by daily screen time (Figure 5C). The dependence plot of serum ferritin (Figure 5D) showed that lower concentration was related to increased probability of ADHD, which established a possible metabolic susceptibility.

In the attempt of holistically quantifying the synergistic effect of the two most important predictors, a joint risk topography map was developed (Figure 5E). This plot was a contour plot of the proportionate contribution of Total IgE and Vitamin D to the SHAP. The topography clearly delineated a ‘storm eye’ of maximum joint risk located in the quadrant representing severe IgE elevation (>500 IU/mL) and extreme Vitamin D loss (<20 ng/mL).

Giving these multidimensional interactions a clinical picture the patient population was dichotomized into four sub-groups by the critical thresholds determined (IgE > 250 IU/mL and Vitamin D < 20 ng/mL). The clinical risk stratification analysis (Figure 5F) showed that the contribution to the joint risk among patients in the “Dual High Risk” group was exponentially compounded and very high as compared to the contribution to the joint risk of the patients in the “Normal / Low Risk” population and the single hazard groups (*p* < 0.001). The existence of such a strong stratification highlights the importance of treating combined clinical profiles, but not independent biomarkers when determining the risk of ADHD comorbidity in children.

### 3.6 Clinical translation, individualized prediction, and risk triage

To ascertain the practical utility of the XGBoost model in a clinical setting, we evaluated its net clinical benefit and translated the complex algorithmic outputs into accessible clinical tools (Figure 6).

**Figure 6:**
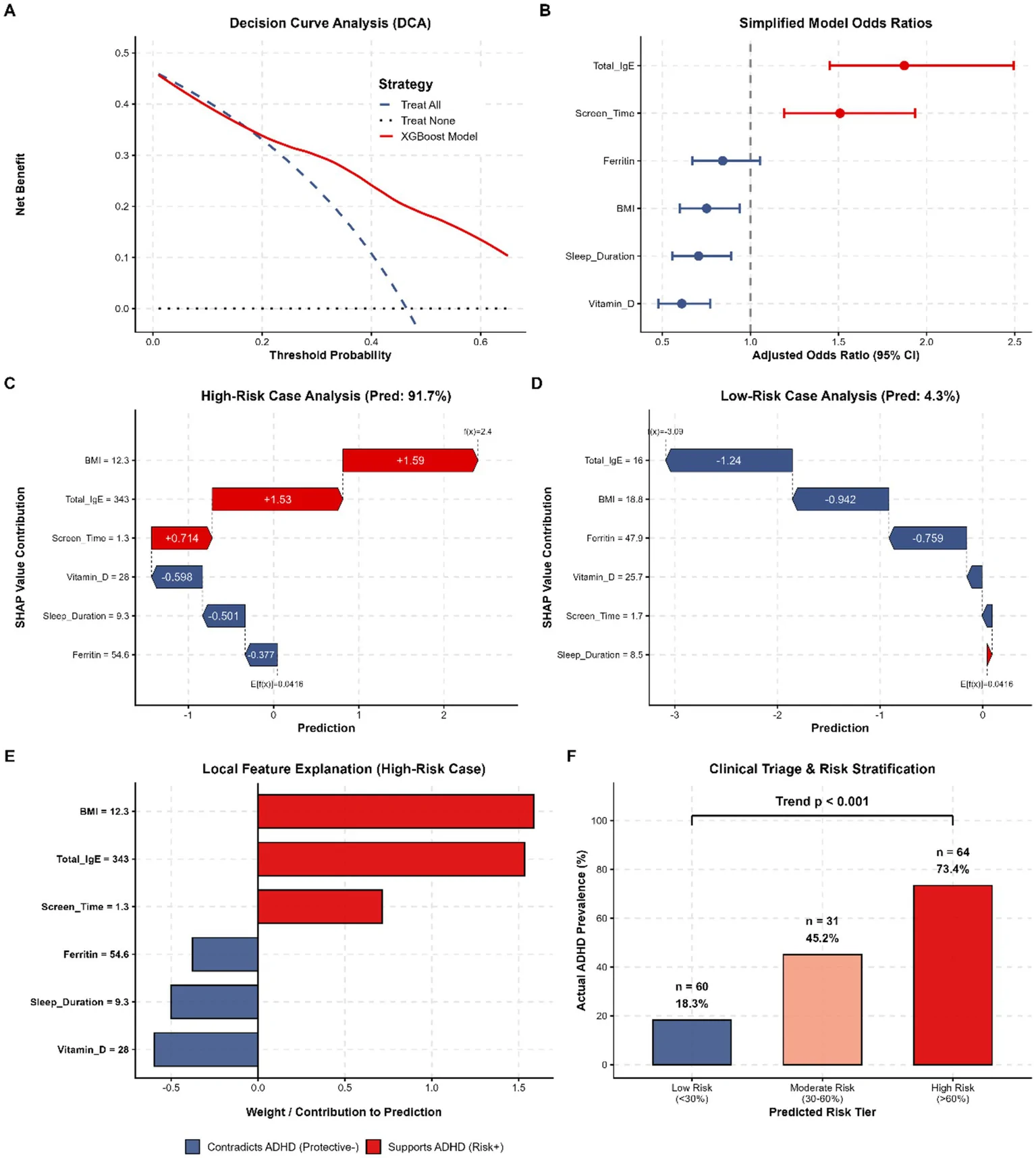
Clinical translation, individual case prediction, and risk triage strategy. **(A)** Decision Curve Analysis (DCA) demonstrating that employing the XGBoost model provides a higher net clinical benefit compared to “treat-all” or “treat-none” strategies across clinically relevant threshold probabilities. **(B)** A simplified forest plot displaying adjusted Odds Ratios (95% CI) derived from a multivariable logistic regression, serving as an intuitive reference for clinicians. **(C,D)** Local SHAP waterfall plots dissecting the prediction trajectories for a representative high-risk patient (Pred: 91.7%) and a low-risk patient (Pred: 4.3%), where red bars indicate risk-promoting factors and blue bars indicate protective factors. **(E)** A summarized local feature explanation bar chart for the high-risk case. **(F)** A proposed clinical triage strategy demonstrating actual ADHD prevalence escalating in a step-wise manner across model-predicted risk tiers (Trend *p* < 0.001), validating its utility for outpatient screening.

A Decision Curve Analysis (DCA) was conducted on the independent test to compare the clinical outcomes of using the predictive model versus using default strategies (i.e., treating all patients or treating none). The DCA curve revealed that, the XGBoost model produced a significantly better net benefit over clinically relevant broad range of threshold probabilities (0.01 to 0.65) than the default approaches (Figure 6A), and this suggests that clinical interventions guided by this model could effectively reduce unnecessary evaluations whilst capturing the actual comorbid cases.

In order to close the gap between machine learning and traditional epidemiological interpretation, a forest plot with the same fundamental features (Figure 6B) was presented by using a multivariable logistic regression. This simplified model provided adjusted Odds Ratios (OR) that were found to support the SHAP results: Total IgE (OR = 1.87, 95% CI: 1.45–2.49) and Screen Time (OR = 1.51, 95% CI: 1.19–1.93) were observed to be major risk-enhancing factors, whereas Vitamin D (OR = 0.61, 95% CI: 0.48–0.77) and Sleep Duration (OR = 0.71, 95% CI: 0.56–0.89) exhibited protective effects.

Individual patient-level clinical applicability of the XGBoost model was also indicated by showing the SHAP waterfall plots. Extreme values of BMI (12.3 kg/m ^2^) and Total IgE (343 IU/mL) acted as profound risk-promoting factors (red bars) in a representative high-risk patient (Predicted probability = 91.7%, Figure 6C), which altered the ADHD comorbidity prediction significantly. On the other hand, the low-risk example (Predicted probability = 4.3%, Figure 6D), with a normal IgE (16 IU/mL) and with enough Vitamin D (25.7 ng/mL) had to accumulate numerous protective SHAP contributions (blue bars), which in turn inhibited the predicted risk. The summary table of directional effect of each clinical indicator on the high-risk individual took another, straightforward form in the shape of the localized feature explanation chart (Figure 6E).

Lastly, model-predictive stratification of the test cohort into three tiers [Low Risk (less than 30%), Moderate Risk (less than 60%), and High Risk (more than 60%)] was done to facilitate rapid clinical triage in pediatric outpatient settings. Figure 6F illustrates that real prevalence of ADHD rose strictly in step-wise pattern in these tiers as the low-risk group, having prevalence of 18.3% and the high-risk group, having prevalence of 73.4% (Trend *p* < 0.001). This large risk of stratification indicates that the model can be directly involved in clinical workflow to make previous multidimensional assessment a priority among highly susceptible pediatric patients.

## 4 Discussion

The co-occurrence of allergic rhinitis (AR) and attention-deficit/hyperactivity disorder (ADHD) in pediatric populations has increasingly garnered clinical attention, primarily driven by emerging evidence of neuro-immune crosstalk (26). Our robust XGBoost predictive model, which we developed and validated in the current study using a hybrid feature selection pipeline, achieved an optimal test-set AUC of 0.878. Moreover, using SHAP interpretability analysis, we not only explained non-linear physiological thresholds of these factors but also discovered a high-order synergistic risk due to joint severe allergic inflammation and nutritional deficiency which provides a multidimensional tool of clinical risk stratification.

Our findings indicated that immunological and nutritional indices predominantly predicted ADHD comorbidity. In particular, the global SHAP analysis identified Total IgE as the most critical feature of risk promotion. The following dependence plots showed there was a clear threshold effect, before which ADHD risk increased dramatically when IgE exceeded about 250 IU/mL. Such a finding agrees with the recent epidemiological research in which chronic systemic inflammation, often presenting with protracted histamine discharge and high levels of cytokines in severe atopics, has been hypothesized to be associated with blood–brain barrier alterations that correlate with disrupted neurocognitive functioning (27–29). On the other hand, the effect of sufficient serum Vitamin D was strong protective impact (30). Surprisingly, our interaction topography map showed that there was a joint high-risk phenotype (high IgE and low Vitamin D), which had a synergizing effect on the comorbid risk. This is in tandem with molecular research indicating that Vitamin D not only acts as a neuroprotectant but also plays a critical immunomodulatory role in the down-regulation of Th2-mediated allergic responses (31, 32). Accordingly, the perceived compound risk indicates that single pathway-based clinical interventions may be too little to help the vulnerable sub-population.

An example of a notable methodological point of our study was associated with the choice and meaning of BMI. Although univariable analysis in Table 1 revealed a non-significant baseline difference (*p* = 0.095) for BMI between the control and case groups, the machine learning algorithms robustly prioritized it as a top predictor. This seeming contradiction is internally solved through realization of the drawbacks of linear statistics. In our SHAP dependence analysis, a U-shaped risk profile of BMI was validated, which means that extreme underweight conditions and extreme obesity conditions might be associated with an increased susceptibility to neurodevelopmental comorbidities (33, 34). These two-way extremes were more or less buried in the traditional comparative mean, which emphasizes the improved ability of non-linear tree-based models to detect complex clinical patterns. However, given the retrospective nature of this study, we acknowledge that tree-based algorithms are prone to capturing artifactual splits. Therefore, the link between non-significant baseline BMI variation and primary neuro-immune processes should be interpreted cautiously as a hypothesis-generating correlation rather than an established biological mechanism. Besides, our methodology relied on four representative machine learning architectures (LR, SVM, RF, and XGBoost) covering linear, non-linear margin, parallel bagging, and sequential boosting paradigms, effectively demonstrating the superiority of ensemble methods over traditional clinical linear models. Although other boosting variants exist, XGBoost was prioritized as the definitive predictive model. This choice was driven not only by its robust discriminative capacity but primarily by its seamless and highly optimized integration with the TreeSHAP algorithm, which uniquely enabled the precise computation of high-order SHAP interaction values vital for unveiling the synergistic neuro-immune phenotypes in this study. Furthermore, the optimal decision threshold (0.527) for the XGBoost model was identified by giving precedence to the balanced Youden Index to produce a sensible clinical sensitivity and specificity profile without necessarily demanding an implausible zero-false-positive value.

Although the system has a promising predictive performance and interpretability, this research study is, in fact, limited in a number of ways that should be noted. Primarily, the current findings rely on a single-center retrospective cohort study design, which inherently restricts the external validity and generalisabilities of the model to various ethnic or geographic pediatric groups. Second, we have obtained detailed measures of baseline measures, but the research was not based on longitudinal follow-up measures; therefore, it was impossible to identify the nature over time or causality of changes in IgE/Vitamin D levels and the progressive course of the ADHD symptoms. Third, the predictive framework did not fully control confounding factors of different pharmacological interventions of AR (including long term use of corticosteroid or antihistamine nasal sprays). Furthermore, while severe asthma and eczema were excluded, the intrinsic severity classification of AR itself and the potential interference from mild-to-moderate concurrent allergic conditions could not be exhaustively stratified, which may still partially confound the association between IgE levels and neurodevelopment. Prospective, multi-center studies that include tracking of symptoms over time and stratification of treatment are required to support and optimize the suggested neuro-immune risk algorithms.

## 5 Conclusion

In summary, this research established a predictive XGBoost model capable of identifying the potential risk of comorbid ADHD in children with allergic rhinitis. We combined a hybrid feature selection pipeline with SHAP analysis, which allowed defining a powerful six-feature clinical profile in which Total IgE and Vitamin D could be identified as the main synergistic factors of neurodevelopmental vulnerability. The model demonstrated substantial discriminative capacity while revealing critical non-linear thresholds that traditional statistical methods often obscure. Moreover, the transfer of these algorithmic findings into a clinical risk stratification model provides a useful instrument to facilitate the inclusion of multidisciplinary assessment of a highly susceptible patient by pediatricians in their priorities. Although these findings provide valuable mechanistic and clinical insights into pediatric neuro-immune crosstalk, multi-centric, prospective research studies should be used in stronger research to confirm the generalizability of the predictive model and to identify whether cross-linkage interventions targeting these immunological and nutritional parameters can potentially change in ADHD courses.

## Data Availability

The raw data supporting the conclusions of this article will be made available by the authors, without undue reservation.
