# Supplementary material for: Development and validation of an interpretable machine learning model for predicting ADHD comorbidity in children with allergic rhinitis: a SHAP-based analysis
Source: Front Public Health. 2026 Sep 23;14:1837156. doi: 10.3389/fpubh.2026.1837156 (PMC13642325; doi:10.3389/fpubh.2026.1837156)
Supplement: Supplementary file 1 [file Table_1.DOCX]

**Supplementary Table S1. Comprehensive Predictive Performance and Optimal Decision Thresholds Across All Machine Learning Models on the Independent Test Set**

| **Machine Learning Model** | **Optimal Threshold (Youden Index)** | **AUC (95% CI)** | **Accuracy (%)** | **Sensitivity (%)** | **Specificity (%)** | **F1-Score** |
| --- | --- | --- | --- | --- | --- | --- |
| Logistic Regression (LR) | 0.505 | 0.713 (0.648–0.778) | 69.0 | 45.8 | 89.2 | 0.579 |
| Random Forest (RF) | 0.482 | 0.811 (0.751–0.871) | 76.1 | 70.8 | 80.7 | 0.734 |
| Extreme Gradient Boosting (XGBoost) | 0.527 | 0.878 (0.824–0.933) | 80.6 | 77.8 | 83.1 | 0.789 |
| Support Vector Machine (SVM) | 0.446 | 0.814 (0.756–0.872) | 74.2 | 77.8 | 71.1 | 0.737 |

Abbreviations: AUC, Area Under the Receiver Operating Characteristic Curve; CI, Confidence Interval.

Notes: The optimal decision threshold for each model was uniformly determined by maximizing the Youden Index (Sensitivity + Specificity - 1) on the test cohort to ensure fair comparative evaluation. Bold values indicate the optimal model (XGBoost) selected for definitive clinical interpretation and SHAP analysis. LightGBM and CatBoost were retrospectively implemented during the peer-review revision process.
